# A Process Optimization and Performance Study of Environmentally Friendly Waste Newspaper/Polypropylene Film Layered Composites

**DOI:** 10.3390/ma13020413

**Published:** 2020-01-16

**Authors:** Neng Guan, Chuanshuang Hu, Litao Guan, Weiwei Zhang, Hong Yun, Xiaojing Hu

**Affiliations:** 1 College of Materials and Energy, South China Agricultural University, Guangzhou 510642, China; 2 College of Forestry and Horticulture, Xinjiang Agricultural University, Urumqi 830025, China

**Keywords:** old newspaper, polypropylene, laminated, composites, mechanical properties

## Abstract

Waste newspaper are currently used in a single way and have low utilization rates. In this paper, the optimal process of preparing environmentally friendly layered composites by using waste newspaper combined with polypropylene film lamination was studied. The effects of hot-pressing temperature, hot-pressing time and paper content on the properties of the composites were analyzed. The results showed that under the process conditions of hot-pressing temperature 180 °C, compression time 20 min and paper content 66.7%, the obtained composite material had a flexural strength of 126 MPa, a tensile strength of 95 MPa, an impact strength of 5.3 kJ/m^2^ and a water absorption thickness expansion ratio of 3.2%. Tensile performance increased by 164% compared to the original waste newspaper. Compared to our previous work, the hot processing time had been cut in half and costs were lower. In terms of creep properties, the unrecoverable strain rate was reduced by 57.5% compared to pure polypropylene. The results show that the material can maintain excellent flexural strength, tensile strength and water absorption performance while making good use of waste newspaper.

## 1. Introduction

As environmental issues are increasingly concerned and the scarcity of petroleum resources, people are increasingly interested in the use of waste, and many wastes can be used as input materials for the development of other industrial new products [1], such as waste paper. China is the world’s largest waste paper producer and consumer country [2]. In 2017, there were 2800 Chinese paper and paperboard manufacturers, and China’s paper and paperboard production was 111.3 million tons, with a consumption of 108.97 million tons [3]. Making full use of waste paper will alleviate environmental problems and resource shortages, and bring huge economic benefits [4,5]. However, the construction of waste paper recycling, sorting, storage and transportation system in China is still relatively backward, with a low industrialization level, and the quality of waste paper recycling is poor. As a result, some waste paper is still degraded, leading to fewer waste paper utilization ways [6] and low utilization rate [7]. In May 2016, the US Forestry and Paper Association announced that the recycling rate of waste paper reached 66.8% [8], 18.7% higher than China. Therefore, in addition to improving China’s waste paper recycling system, it is increasingly important to find a way to use waste paper efficiently. The waste paper is rich in fiber, and there are a large number of polar hydroxyl groups on the surface of the paper fiber. It also has the similar structure and properties of natural plant fibers. Compared with natural plant fibers and synthetic fibers, they have high consistency of quality, fiber length, source and mechanical properties and are readily available [9,10,11].

In the waste paper recycling route, the more primitive ways are composting [12], burning and landfill. However, waste paper containing aluminum sulfate, caustic soda and other paper accessories, which will cause environmental pollution [13]. Recycled paper is a highly utilized product, but the added value of the product is low and poses a threat to the environment [14]. In recent years, researchers at home and abroad have carried out a lot of work. For example, carboxymethyl cellulose and cyanoethyl cellulose [15,16,17,18] were synthesized using waste paper. Paper fiber composites have also been prepared using waste paper as a raw material. This required smashing the newspaper and then mixing it with resin and then compounding it by extrusion molding [19,20,21,22]. The strength of the obtained material was comparable to that of the glass fiber composite. However, there are also many problems in the process, such as paper fiber tends to become flocculent after comminution, which increases the difficulty of melting and blending with the polymer matrix, and results in uneven dispersion of paper fiber in the matrix. Our research team has found that old newspaper can solve the flocculation problem with low-dose gaseous methyltrichlorosilane (MTCS) treatment [23]. Some scholars had also investigated the impact of deinking methods of waste newspapers on the properties of composite materials [24]. In addition, the interface between the fiber and the matrix is an important factor affecting the properties of the polymer composite [25,26]. The interaction between the hydrophilic fiber and the matrix is usually limited, while the matrix is usually hydrophobic, which leads to poor interfacial adhesion, limiting mechanical properties and affecting long-term performance at the same time. In sisal fiber (SF) reinforced HDPE composites prepared by extrusion mechanism, Xuefeng Zhao et al. explored the influence of fiber content interfacial compatibility and preparation process on its mechanical properties (tensile, impact and creep). It was found that the increase of fiber content and the compatibility of the interface with maleic anhydride grafted HDPE (MAPE) can improve the mechanical properties of the composite [19]. F. Vilaseca et al. prepared polypropylene (PP) composites from kraft fiber recovered from old bags and used Maleic anhydride grafted polypropylene (MAPP) as a coupling agent to improve the compatibility and adhesion of the fiber matrix [27]. A. Serrano et al. not only added MAPP to prepare composite materials, but also used the micromechanical and mechanical models to inversely calculate the Young’s modulus inherent in composite materials [28]. But this kind of wood-plastic composite (WPC) all needs to destroy the paper fibers and blend them, which undoubtedly increases the difficulty and cost of production.

In recent years, fiber composite materials prepared by hot pressing have emerged. This method avoids the damage to the fiber, further improves the mechanical properties, and breaks through the limitation of the size of the product prepared by extrusion and extrusion process [29]. Prambauer Martina et al. used newspapers, copy paper, filter paper as raw materials, supplemented with compatibilizer maleic anhydride grafted polypropylene, respectively cross-laminated with polypropylene film and then hot-pressed to prepare paper-plastic laminated composite. The results showed that the good mechanical strength of composites prepared using copy paper and newspaper respectively, and when the volume content of paper was 30%, the tensile and flexural strength of copy paper/PP laminated composites was the highest, about 85 MPa and 90 MPa [30,31]. As well as our previous research [32], high-strength laminates were prepared using newspaper layer as a reinforcing material and high density polyethylene (HDPE) film as the matrix. Such composite materials still need to be improved in terms of mechanical properties and processing efficiency. In addition, most of the research has only been carried out in theoretical scenarios, has not been linked with real products, and has not brought actual economic benefits.

In this study, it is attempted to explore the best preparation technology and mechanism by using the original waste newspaper and polypropylene film laminating compound, and obtain the composite material with better physical and mechanical properties, and the processing efficiency is further improved while achieving or exceeding the performance of similar materials. The composite material not only maintains the original shape of the newspaper, improves the utilization rate of waste paper, but also has a simple processing method, green environmental protection and recyclability. On the other hand, the modified high-performance composite materials can be applied to high value-added products such as sports skateboards and laminate flooring, which can not only turn waste into treasure, but also fully utilize resources and reduce the serious environmental pollution problems. 

## 2. Materials and Methods

### 2.1. Materials

The used newspapers (272 mm × 390 mm × 0.055 mm, weight: 47.4 g/m^2^) were recycled from Shanghai Securities News. The content of cellulose, hemicellulose and lignin were as follows: 52.59%, 26.91%, 15.60%. The tensile strength of the newspaper was 39.5 MPa. Polypropylene film (density 0.925 g/cm^3^, MFI 11 g/10 min at 190 °C /2.16 kg, length and width 272 mm × 390 mm) was purchased from Huizhou Bao tian Plastic Packaging Co., Ltd., Huizhou, China. The tensile strength measured was 35 MPa, the thickness was selected to be 0.022 mm, 0.025 mm, 0.03 mm, 0.04 mm, 0.055 mm.

### 2.2. Preparation Method of Composite Materials

The newspaper and polypropylene film were assembled in the order of a newspaper and a polypropylene film, a total of 108 layers. The assembled composite material was dried in a drying box at 60 °C until the quality was constant. A hot press (XLB-600 × 600, Qingdao Goworld Rubber Machine Co., Ltd, Qingdao, China) was used to press the assembled composite material. The parameters were shown in Table 1. Among them, the paper content (wt.%) was controlled by using polypropylene films of different thicknesses. The thickness of the product was controlled by the thickness gauge to 3 mm. At the same time, in order to prevent the surface of the composite material from being contaminated, a PTFE film was laid on the upper and lower surfaces. After the hot-pressing process was finished, the composite material was cooled to room temperature using a circulating cooling water system and then the pressure was released.

After the above process was completed, the pressure was unloaded, and the sample was taken out and equilibrated at 23 °C ± 2 °C, humidity 50% ± 5 for at least 24 h.

### 2.3. Composite Material Performance Test Method

The tensile strength of the original waste paper was tested in accordance with ASTM D828-16. Universal testing machine (ai-7000m-go, high-speed railway testing instrument (Dongguan) co., LTD., Dongguan, China) was used to conduct 10 parallel tests on each group of samples, with a gauge length of 180 ± 5 mm and a test speed of 10 mm/min, to ensure that the samples broke in 10–30 s.

The flexural strength of the composite material was tested in accordance with GB/T 17657-2013. The specimen size was 150 mm × 5 mm × 3 mm and the test speed was 25 mm/min. Tensile strength was tested with reference to the first type of specimen in ASTM D638-14 at a test speed of 5 mm/min. The gauge length was 50 mm. Izod impact strength was referenced to ATSM D256-2010 using an electronic cantilever pendulum impact tester (XJUD-5.5, Chengde Jinjian Testing Instrument Co., Ltd., Chengde, China). The 24 h water absorption thickness expansion rate test was carried out according to GB/T 17657-2013.

The creep of the sample was measured using a dynamic thermomechanical analyzer (DMA 242E Artemis, NETZSCH-Gerätebau GmbH, Selbu, Germany) with a sample size of 55 mm × 8 mm ×3 mm. A load of 0.42 MPa was applied to the sample, creep behavior was observed within 40 min, then the load was released within 40 min and the recovery behavior was observed.

Scanning electron microscopy (SU-70, HITACHI, Tokyo, Japan) with an acceleration voltage of 2.0 kV was used to observe the cross-section of the composites under different process conditions. Before the observation, all the sections were sprayed with gold.

## 3. Results and Analysis

According to the experimental arrangement of Table 1, the three factors of temperature, time and paper content were carried out. Then the physical and mechanical properties of the composite were tested. Through the multivariate analysis of variance of the test results, the results showed that the hot-pressing temperature and paper content had a significant impact on the properties of the composite materials, while the hot-pressing time was not significant, as shown in Table 2.

### 3.1. Effect of Hot-Pressing Temperature on the Properties of Composites

**Figure 1:**
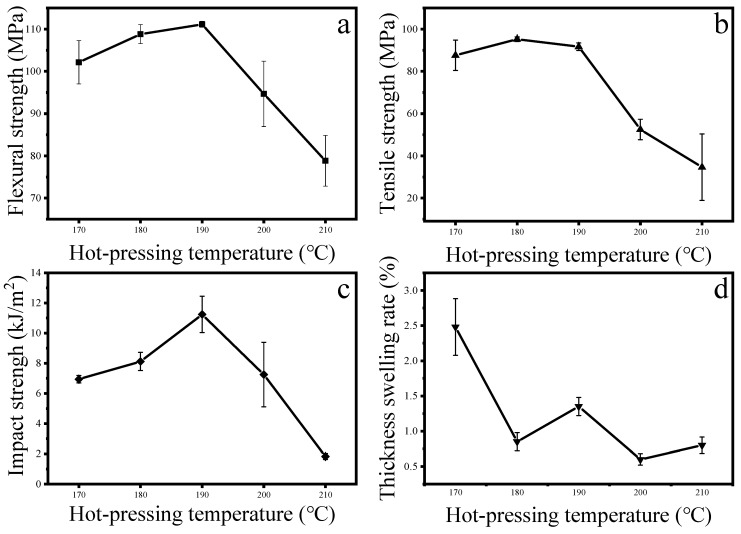
Effect of hot-pressing temperature on different properties of composite plates: (**a**) flexural strength; (**b**) tensile strength; (**c**) impact strength; (**d**) thickness swelling rate.

The strong interfacial bonding of composite materials involves factors such as mechanical riveting and making fibers fully wet [33]. As shown in Figure 1, the effect of temperature on the properties of the composite material was significant. When the hot-pressing temperature rose from 170 °C to 190 °C, the flexural strength, tensile strength and impact strength of the composite board reached 111 MPa and 92 MPa, 11.2 kJ/m^2^, respectively, with corresponding increases of 8.8%, 4.6% and 61.9%. The expansion rate of 24 h water absorption thickness was 1.35%, which decreased by 45.6%. This is because the polypropylene melts more fully with the gradual increase of temperature. Under the action of pressure, the fluidity is enhanced, which can penetrate into the pores of the newspaper better and strengthen with the mechanical riveting of the newspaper. This explanation was also mentioned in related articles [22]. When the hot-pressing temperature was increased from 190 °C to 210 °C, the material properties began to decrease. The flexural strength, tensile strength, impact strength and 24 h water absorption thickness expansion ratio of the composite sheet were 79 MPa, 35 MPa, 1.8 kJ/m^2^ and 0.80% respectively, correspondingly reduced by 29.1%, 62.2%, 83.7%, and 40.7%. This is mainly due to the large amount of oxidation, dehydration and β-alkoxy elimination of cellulose at high temperatures, and the higher the temperature, the faster the degradation rate of cellulose [34].When the hot-pressing temperature reached 200 °C, the functional groups which are easily oxidized by hydroxyl groups on the paper fibers are rapidly oxidized, the number of hydrogen bonds formed between the molecules is reduced, and the binding force between the molecules is lowered, resulting in a decrease in the performance of the newspaper substrate. The 24 h water absorption thickness expansion rate was always in a lower state after 180 °C. the increase in temperature is more conducive to the melting of polypropylene and penetration in newspaper, thus having a better embedding effect on the newspaper layer. Therefore, 180 °C was selected as the experimental temperature in the following experiments.

### 3.2. Effect of Hot-Pressing Time on the Properties of Composites

**Figure 2:**
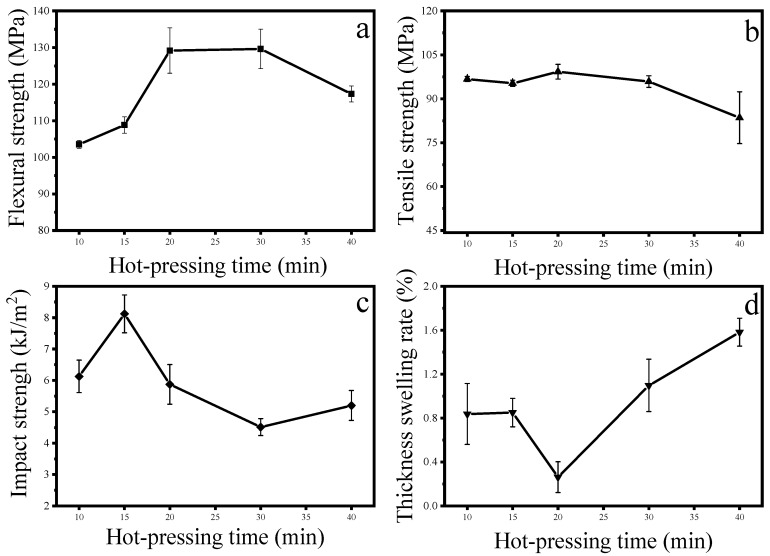
Effect of hot-pressing time on the performance of composite panels: (**a**) flexural strength; (**b**) tensile strength; (**c**) impact strength; (**d**) thickness swelling rate.

During the hot-pressing process, the heat in the plate is transferred from the upper and lower plates to the center layer of the composite material. The heat transfer speed is determined by the hot -pressing temperature and the hot-pressing time. When the hot-pressing temperature was 180 °C and the hot- pressing time was increased from 10 min to 20 min, the flexural strength and tensile strength of the composite material reached 129 MPa and 99 MPa, respectively, which were 24.8% and 2.6% higher than 10 min, respectively. This is because increase in hot-pressing time at a certain temperature can make the polypropylene melt more fully and penetrate the newspaper better. In the pores, a riveting structure is formed to achieve a better bonding effect between polypropylene and the paper layer. However, when the hot-pressing time was increased from 20 min to 40 min, the flexural strength and tensile strength of the composite panel were changed to 117 MPa and 84 MPa respectively, which were reduced by 9.2% and 15.8%. This is because the excessive heating time caused the newspaper to oxidize, resulting in a decline in the performance of the newspaper itself. Oxidation and decomposition occur in paper fibers under heating and pressure conditions of 180 °C. Although the degree is not high, the strength decreases with time. Therefore, the flexural strength and tensile strength in Figure 2 have a tendency to decrease with time, but they are not as severe as those in Figure 1. The excessively long hot-pressing time causes the hydrophilic hydroxyl groups on the paper fibers to be partially oxidized, resulting in weakening of the intermolecular forces, and water molecules are more likely to enter the matrix, and the water absorbing thickness expansion rate is increased. Similarly, the excessive heating time reduced the impact strength. From 10 min to 15 min, the impact strength increased by 32.5%, but then began to decrease. During the experiment, the composite paper turned yellow and became brittle, which also illustrated the above points.

### 3.3. Effect of Paper Content on the Properties of Composites

**Figure 3:**
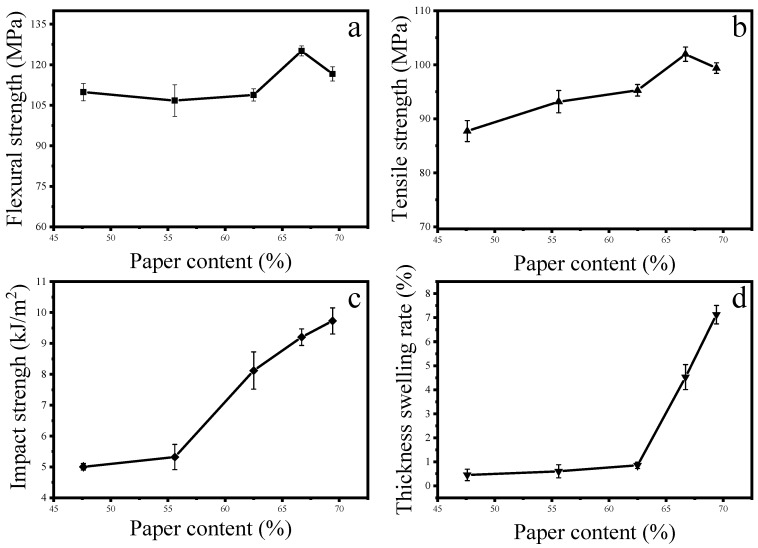
Effect of paper content on the properties of composites: (**a**) flexural strength; (**b**) tensile strength; (**c**) impact strength; (**d**) thickness swelling rate.

As shown in Figure 3, the flexural strength, tensile strength and impact strength of the composites increased with the increase of the paper content, and then decreased, because the plant cellulose rigidity is much larger than that of the pure polypropylene [35]. According to the law of mixing, the total mechanical properties of the two increase with the increase of paper content. However, when the paper content was higher than 66.7%, the mechanical properties were degraded. In the composite material, the newspaper fiber plays a major mechanical strength, and the polypropylene content is too small to sufficiently penetrate and rivet the paper fiber, so the mechanical properties are degraded.

The influence of paper content on the swelling ratio of water absorption thickness of composite materials was very significant. When the paper content was more than 62.5%, the swelling of water absorption thickness increased rapidly. This is due to the gradual reduction of polypropylene content and mechanical riveting cooperation. With the weakening, the exposed hydroxyl groups also increase, and the water absorption thickness expansion rate increases. In addition, the four sides of the test piece were not edge-sealed in the experiment, so the water absorption problem could be improved by the edge-sealing treatment in actual use. According to the above results, too high or too low paper content will have adverse effects on composite materials.

### 3.4. Creep Performance

**Figure 4:**
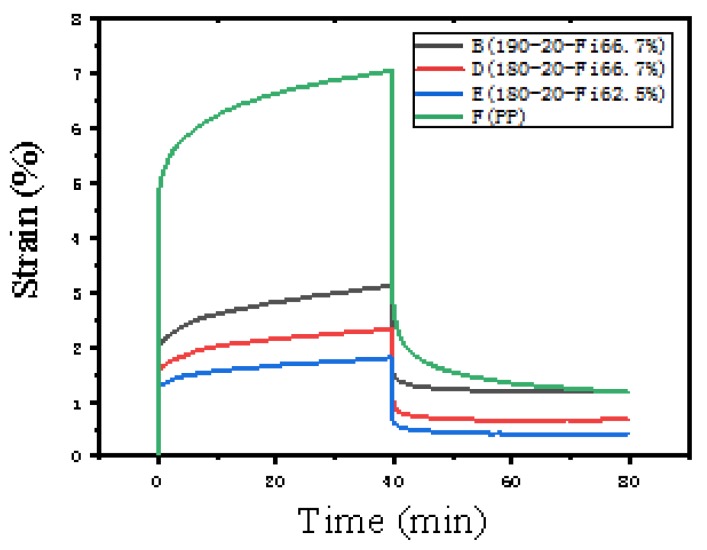
Creep curve of composite material and polypropylene: (B) the hot-pressing temperature was 190 °C, the hot-pressing time was 20 min, and the paper content was 66.7%; (D) the hot-pressing temperature was 180 °C, the hot-pressing time was 20 min, and the paper content was 66.7%; (E) the hot-pressing temperature was 180 °C, the hot-pressing time was 20 min, and the paper content was 62.5%; (F) the hot-pressing temperature is 180 °C and the hot-pressing time is 20 min.

Figure 4 shows the creep properties of composites at different temperatures (180 °C, 190 °C) and different paper contents (62.5%, 66.7%). In the process of loading a constant force, polypropylene was most deformed compared to composite materials because the rigidity of cellulose in newspaper is greater than that of pure polypropylene [35]. The rise in the hot-pressing temperature increased the deformation of the composite material. The higher the temperature, the greater the degree of oxidation of the paper fibers, resulting in a decrease in mechanical strength. Therefore, the deformation of B was greater than D. At the same hot -pressing temperature, too little polypropylene makes it not penetrate well into the pores of the newspaper and forms a coating, which increases the deformation of the material, as shown in D and E in Figure 4. After the load was removed, the irreversible deformation of the polypropylene was the largest, reaching 1.17%. The composite material, due to the mechanical riveting between the polypropylene and the newspaper, limits the slip of the polypropylene molecular chain and reduces the irreversible deformation. When the hot-pressing temperature was 180 °C and the paper content was 62.5%, the irreversible shape of the composite material became 0.415%, which was the non-reversible shape minimum value.

### 3.5. Microscopic Morphology of Composite Materials

**Figure 5:**
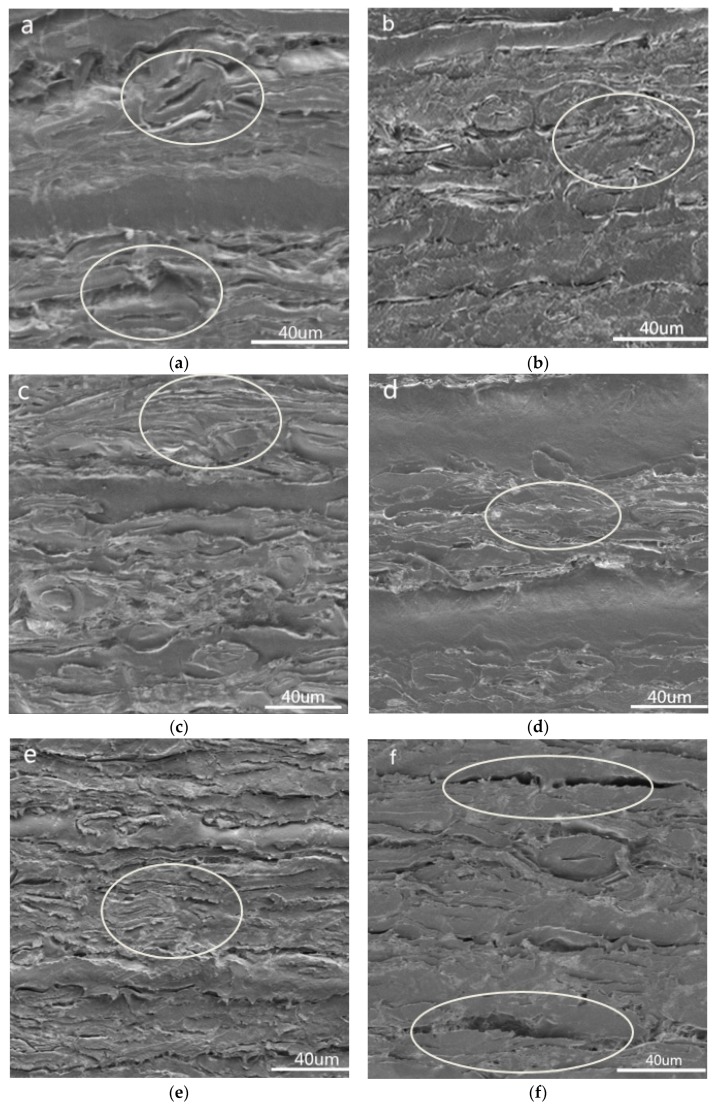
Microscopic morphology after magnifying 500 times of composite materials at different temperatures and different paper contents: (**a**) hot-pressing temperature: 170 °C; (**b**) hot pressing temperature: 190 °C; (**c**) hot-pressing temperature: 200 °C; (**d**) paper content: 47.6%; (**e**) paper content: 62.5%; (**f**) paper content: 69.4%.

The microstructure of the composites at different temperatures and different paper contents was observed by SEM. As shown in Figure 5a–c, the hot-pressing temperature had a large effect on the permeability of polypropylene. At 170 °C, there was a large amount of porosity in the composite paper fiber layer (Figure 5a, and the effect of pressure did not completely press the paper fibers to compact. As the temperature increased, the polypropylene penetrated better into the pores of the newspaper (Figure 5b,c). When the temperature reached 190 °C or higher, the gap between the newspaper fibers was substantially filled. The melting point of polypropylene is 162.12 °C, so the increase in temperature contributes to the flow of polypropylene. As shown in Figure 5d–f, the influence of paper content on the penetration of polypropylene was not obvious. When the paper content increased from 47.6% to 62.5%, SEM of the composite cross section showed that there were relatively uniform and complete polypropylene penetration and adhesion in the paper layer. Natural fibers are hydrophilic and polypropylene is hydrophobic, which prevents good compatibility between the two phases [36]. When the paper content was more than 62.5%, the reduction of polypropylene exacerbated this phenomenon, and a small amount of voids appeared between the layers (Figure 5f). Thus, excessively high paper content adversely affected the mechanical properties of the composite, and this result was consistent with 3.3.

## 4. Conclusions

In this study, environmentally friendly paper-plastic laminated composite panels were prepared by using waste paper and polypropylene film as raw materials, and the effects of hot-pressing process parameters and paper content on the properties of layered composite sheets were studied. Through single factor experiments, it was found that hot-pressing temperature and paper content had significant effects on the mechanical properties of composites. Too high temperature will make paper fiber oxidation, too low temperature will be not conducive to the flow and penetration of polypropylene. Therefore, the optimum process for determining the newspaper/polypropylene layered composite material was hot-pressing temperature of 180 °C, hot-pressing time of 20 min, and paper content of 66.7%. The tensile strength and flexural strength of the obtained composite material were nearly three times higher than polypropylene, and the creep resistance was nearly doubled than polypropylene. The utilization rate of newspaper was further improved. Compared with the method of crushing newspapers for extrusion production [23], the flexural strength and tensile strength of the obtained materials had been increased by nearly 260% and 188%, respectively (the performance value under the optimal conditions of each process). It is shown that the use of the entire sheet of paper can help to improve some of the mechanical properties of the composite material. Compared to our previous work [32], the hot processing time had been cut in half and costs were lower. The composite material is of great significance for promoting the recycling of waste paper and improving the performance of polymer materials.

## Figures and Tables

**Table 1:** Single factor test table.

Hot-Pressing Temperature (°C)	Hot-Pressing Time (min)	Paper Content (%)
170, 180, 190, 200, 210	15	62.5
180	10, 15, 20, 30, 40	62.5
180	15	69.4, 66.7, 62.5, 55.6, 47.6

Note: The hot-pressing pressure used was 1 MPa.

**Table 2:** Analysis of variance.

Variation Sources	df	Flexural Strength			Tensile Strength			Impact Strength			Thickness Swelling Rate of Water Absorption (24 h)		
		MS	F	Sig.	MS	F	Sig.	MS	F	Sig.	MS	F	Sig.
Hot-pressing Temperature	4	380.591	3.938	0.036	1160.629	41.945	0.00	11.246	3.322	0.056	0.700	0.157	0.955
Hot-pressing Time	4	247.95	1.656	0.236	94.128	0.207	0.929	4.016	0.640	0.646	0.920	0.211	0.926
Paper content	4	99.128	0.474	0.754	134.752	0.308	0.866	5.012	0.853	0.524	10.959	31.562	0.000

Note: df is degree of freedom and MS is mean square; F is the statistic; Sig. < 0.5 means significant.
